# Pilot survey of oral health-related quality of life: a cross-sectional study of adults in Benin City, Edo State, Nigeria

**DOI:** 10.1186/1472-6831-5-7

**Published:** 2005-07-25

**Authors:** Christopher Okunseri, Amit Chattopadhyay, R Iván Lugo, Colman McGrath

**Affiliations:** 1 Department of Clinical Sciences, School of Dentistry, Marquette University, Milwaukee, P.O. Box 1881, Wisconsin, 53201. USA; 2 Department of Dental Informatics, Temple University School of Dentistry, Philadelphia, USA; 3 Department of Periodontology and Public Health, Faculty of Dentistry, Prince Philip Dental Hospital, University of Hong Kong, Hong Kong

## Abstract

**Background:**

Oral health studies conducted so far in Nigeria have documented prevalence and incidence of dental disease using traditional clinical measures. However none have investigated the use of an oral health-related quality of life (OHRQoL) instrument to document oral health outcomes. The aims of this study are: to describe how oral health affects and impacts quality of life (QoL) and to explore the association between these affects and the oral health care seeking behavior of adults in Benin City, Edo State, Nigeria.

**Methods:**

A cross-sectional survey recruited 356 adults aged 18–64 years from two large hospital outpatient departments and from members of a university community. Closed-ended oral health questionnaire with "effect and impact" item-questions from OHQoL-UK^© ^instrument was administered by trained interviewers. Collected data included sociodemographic, dental visits, and effects and impact of oral health on QoL. Univariate and bivariable analyses were done and a chi-square test was used to test differences in proportions. Multivariable analyses using ANOVA examined the association between QoL factors and visits to a dentist.

**Results:**

Complete data was available for 83% of the participants. About 62% of participants perceived their oral health as affecting their QoL. Overall, 82%, 63%, and 77% of participants perceived that oral health has an effect on their eating or enjoyment of food, sleep or ability to relax, and smiling or laughing, respectively. Some 46%, 36%, and 25% of participants reported that oral health impact their daily activities, social activities, and talking to people, respectively. Dental visits within the last year was significantly associated with eating, speech, and finance (P < 0.05). The summary score for the oral health effects on QoL ranged from 33 to 80 with a median value of 61 (95% CI: 60, 62) and interquartile range of 52–70. Multivariable modeling suggested a model containing only education (F = 6.5, pr>F = 0.0111). The mean of effects sum score for those with secondary/tertiary education levels (mean = 61.8; 95% CI: 60.6, 62.9) was significantly higher than those with less than secondary level of education (mean = 57.2; 95% CI: 57.2, 60.6).

**Conclusion:**

Most adults in the study reported that oral health affects their life quality, and have little/no impact on their quality of life. Dental visits within the last year were associated with eating, speech, and finance.

## Background

Although common oral diseases are not life threatening, their outcomes may influence the overall wellbeing of individuals and populations. Oral health-related quality of life (OHRQoL) characterizes a person's perception of how oral health influences an individual's life quality and overall well being. This concept has received a lot of attention in the past two decades from sociologists, psychologists and the health professions, [1-15] with different instruments been developed to measure quality of life (QoL) and OHRQoL.

Cohen & Jago [2] first recognized the lack of data on the psycho-social impact of oral health problems. [3] To address this, several authors developed socio-dental indicators to measure the social impact of oral health problems. [5-10] In addition, other generic and disease-specific measures were developed based on the conceptual framework of the World Health Organization's (WHO) International Classification of Impairments, Disabilities and Handicaps (ICIDH). [1,6-16] However, some concerns have been raised about the instruments so far developed, because of their use of older adults in testing the reliability and validity of the instruments, the use of non-random samples, and some have mostly professionally dominated opinions. [13,15] Other concerns also include measuring of positive and or negative effects related to QoL and the varied number of item questions or domains in each instrument. [13,15]

Most of the OHRQoL instruments developed so far measure either the "effect" or the "impact" of oral health on life quality and others measure the "effect and "impact" together. The "effect" dimension examines the physical, psychological and social effects of oral health attributes, and the "impact" dimension examines the impact of oral health attributes on daily activities, chewing ability and talking to people. It also examines the impact of the effects on individuals' overall quality of life. This "effect" and "impact" domains of oral health are better assessed using OHRQoL measures rather than the traditional clinical disease status measures. Slade & Spencer [8] and Adulyanon *et al*. [12] instruments for the most part focused on the negative effects of how oral health affects quality of life, but that developed by McGrath & Bedi [13] included the positive "effects" dimensions which reflected the concept of health beyond the mere absence of disease-impairment-disability-handicap. [13] Developing this idea further, Locker [17] suggested an extension of the ICIDH scope to include certain feeling states (e.g., pain and psychological discomfort) which are prominent consequences of oral disease.

The instrument (OHQoL-UK^©^) developed by McGrath & Bedi used a random probability sampling method. [13,15] It is based on the public's perceptions in the United Kingdom of how oral health affects life quality. [13,15] It consists of 16 key questions relating to 16 key areas of oral health-related quality of life, such as comfort, speaking, and social life, and each of the 16 key questions are also rated for their 'impact' on overall quality of life. [13,15] OHQoL-UK^© ^has been tested for reliability and validity and found to be a valid and reliable measure for assessing OHRQoL, and have also been reported to have good psychometric properties. [13]

OHQoL-UK^©^ and other oral health-related quality of life instruments have been used to explore a relationship between sociodemographic factors in different populations, [18-20] from different countries including Tanzania, Greece, Thailand, Germany, Syria, Egypt, Saudi Arabia, and Uganda. [12,18-22] This has lead to a paradigm shift from the use of only traditional assessment of oral health with a focus on disease to a more comprehensive community measure of health service provision. [1] This shift gives healthcare providers the opportunity to move from the concept of just treating disease, to a holistic model of caring for the patient as a productive member of the society under the "socio-environmental-medical model" of caregiving that encompasses a broader definition of oral health.

Studies show that OHRQoL is related to age, gender, and socioeconomic factors. [6,22] A study of secondary school students conducted in Nigeria found that participants perceived their teeth to be important for their appearance [23] and self esteem. [24] Overall, their perception of the importance of dental health was similar to those reported from the United States. [25] In a recently published study conducted in Nigeria we demonstrated that being younger, being female, and being employed were associated with visiting a dentist in the past 12 months. [26] Other studies have documented prevalence of dental caries and periodontal disease in Nigeria, [27] and described oral health care practice among physicians, [28] as well as oral health knowledge and attitudes of Nigerian school teachers. [29]

Despite these studies there is a paucity of information on how oral health affects and impacts quality of life of persons from sub-Saharan African countries (e.g., Nigeria) which have multiple tribes, varied cultural beliefs, high levels of unemployment and poverty. The specific aims of this study were: 1) to describe the effect and impact of OHRQoL factors, and 2) to explore the association between these effects and oral health care seeking behavior of adults in Benin City, Edo State, Nigeria. This study used oral health related quality of life measures patterned after OHQoL-UK^© ^[13]. The questions of how oral health is related to quality of life were described in two dimensions: "effects" and "impacts". The effect dimension included three domains (physical effects, psychological effects and social effects), and the impact dimension included three domains (impact on daily activities, chewing ability and talking to people). We believe that this study will fill a gap in OHRQoL on Benin City, Edo State, Nigeria and will serve as an impetus for more research in this area.

## Methods

### Sample selection

This study was conducted in Benin City a town which has a population of 2.2 million. The city is a major commercial center that serves as the gateway between the northern, western, and eastern parts of Nigeria, and is home to a substantial number of people from all the major Nigerian tribes/ethnic groups. One of the four Nigerian dental schools is located in Benin City. Anecdotal evidence suggests that the general and teaching hospitals located in the city treat the most patients compared to all private clinics in the state put together. The individuals using these hospitals come from all levels of the socioeconomic fabric of the society. The teaching hospital in Benin City is also adjacent to a large university community. Individuals for the most part pay for dental services out of pocket on a fee-for-service basis at government-owned and privately-owned dental clinics.

Four hundred and twenty six persons were recruited to participate in this study, of which three hundred and fifty-six (83%) had complete usable information. Participants aged 18–64 years were recruited from two large outpatient medical care facilities (University of Benin Teaching Hospital and Central Hospital), and from the adjacent university community. Three interviewers were trained by one of the authors (CO). The interviewers conducted face-to face interviews with the adult participants at the waiting area of the medical outpatient clinics over a 5-week period in the summer of 1999. On average, it took 10 minutes of contact time between the interviewer and the participant in the outpatient waiting area to complete one questionnaire. The importance of collecting this data was explained to participants and their participation was strictly voluntary with no incentives offered.

### Data collection

The closed-ended questionnaire was prepared in English and consisted of the 16 key questions of OHRQoL identified in the OHQoL-UK^© ^by McGrath *et al*. 2000. [13,15] The questionnaire was pre-tested among a group of medical hospital outpatients and university students before it was administered to the study participants. The questions of how oral health is related to quality of life was patterned after OHQoL-UK^© ^[13,15,22,30,31] and described in two dimensions "effects" and "impacts".

The effect dimension included three domains (physical effects, psychological effects and social effects), and the impacts dimensions included three impact item questions (impact on daily activities, chewing ability and talking to people). The "impact" item questions used in this study included only 3 item questions from the original OHQoL-UK^©^, and was analyzed separately from the "effect" portion of the instrument. Participants were interviewed using closed-ended questions, such as; "What affect does your oral health have on your eating or enjoyment of food"? Possible responses on the "effect" were: "Very Good, Good, None, Bad, Very Bad". For example, a question on impact was: "Have problems with your teeth or gums affected your daily activities such as your work or hobbies? Possible responses were: all of the time, most of the time, some of the time, little of the time, none of the time. Each item was scored on a Likert scale from 1 to 5, with a "very bad effect" scored as 1, very good effect as 5, and no effect as 3. The sum of individual item responses were added together to generate an overall OHQoL-UK^© ^score with possible values ranging from 16–144. Additionally, the sum of the responses to items in each domain (physical, psychological, and social) produced subdomain scores. Other data collected were age, self-reported oral health problems, dental visits, gender, ethnicity, number of teeth they possessed, and educational status.

### Data analysis

Data from the paper questionnaires were entered into a computer using SPSS v10.0 for Windows [32] and later converted to SAS^® ^data sets (SAS^® ^V 8.2 Cary, NC, USA) for all analyses. [33] The variables available for this study were sex, age, educational level, employment status, tribe/ethnicity, and last dental visit. Education was categorized into two groups: primary education, and secondary/tertiary education. Oral health care utilization variable was derived from the question: how long ago was it since your last visit to the dentist? The possible responses to this question were: "within the last twelve months", "between twelve and thirty six months" and the last option was "never been to a dentist". We dichotomized the variable at the 12-month time point.

Univariate analyses were conducted for all variables, and all missing/out of range values were verified against the paper questionnaire for accuracy and data entry errors corrected. We evaluated bivariable associations of available variables with visit to a dentist in the past 12 months and sex using Chi-square tests. Statistical significance was inferred at *P < 0.05*. For multivariable analyses, visit to dentist in the past one year (yes/no); sex; education (primary, and secondary/tertiary); employment (yes/no) were dichotomized, whereas age was categorized into three levels –18–24 years, 25–34 years, 35+ years; and ethnicity had four levels – Edo, Ibo, Yoruba, Others (the first three being major Nigerian tribal/ethnic groups). First, we assessed whether the differences arising out of the bivariate analyses remained after adjusting for confounding by sociodemographic factors. For this, we used logistic regression models for modeling the association between visits to a dentist in the past year and the OHRQoL measured attributes. Thereafter we assessed in the same way whether the differences between the two sexes remained upon adjustment for confounding by sociodemographic factors.

In another set of multivariable analyses, we assigned positive integer values to the effect responses (from 1 = very bad to 5 = very good) to derive a sum score for effects. Thereafter, we used this sum score of effects as a continuous variable. We evaluated differences between explanatory factor groups using ANOVA models studying main effects only in SAS^® ^employing PROC GLM. For pair-wise comparisons, we employed Scheffe's test, controlling for Type I errors in post hoc testing of differences in group means.

## Results

Eighty six percent of the participants were below 35 years of age; 55% were women; and 88% had secondary/tertiary education. Most participants (71%) reported that they will only visit a dentist when they need treatment and 21% reported that they had current dental problems, but choose to delay getting the required treatment. Some 88% of participants reported that they could not afford dental treatment, and 89% reported that they were not ready to spend money on dental treatment. Overall, 62% reported that they perceived their oral health to be good; 35% as moderate and 3% as bad. The summary score for the oral health effects on quality of life for the participants ranged from 33 to 80 with a median value of 61 (95%CI: 60, 62) and interquartile range of 52 – 70.

Table 1 shows the response distribution for the effect dimension of OHRQoL. Overall, more than 17% of participants reported a good or very good effect of oral health related issues on their quality of life on each of the domains (physical, psychological and social). About 18–47% participants reported that oral health issues did not have any effect on different aspects within each domain (Table 1). In general, the proportion of participants reporting 'no effect' was substantially lower than those reporting a good or very good effect. The only two exceptions to this pattern was the effect of oral health issues on finances and on work. For effect on finances, 18% participants reported bad, 0.8% very bad effect; 44% participants reported no effect; and about 19%, and 17% reported good or very good effect respectively. About 47% of participants reported no effect of oral health on work 26% and 22% reported a good or very good effect respectively.

**Table 1:** Attributes of the "effect" dimension of oral health related quality of life of study populations (n = 356).

		**Response (percent) to "effect" questions**				
		
**Domain**	**Attribute (effect on)**	**Very bad**	**Bad**	**None**	**Good**	**Very good**
**Physical**	Eating	1.4	8.7	18.4	34.4	37.2
	Appearance	0.6	3.1	23.5	37.2	35.8
	Speech	0.6	2.8	26.5	34.9	35.2
	General health	0.28	3.9	22.1	45.5	28.2
	Breath	0.6	6.4	29.6	36.0	27.4
	Comfort/Relaxation	0.3	8.9	31.2	32.7	26.8

**Psychological**	Sleep	1.4	4.8	36.6	27.4	29.9
	Confidence	0.3	6.7	29.6	32.1	31.3
	Worry	1.1	3.6	41.9	29.3	24.0
	Mood	0.6	6.15	44.4	27.9	21.0
	Personality	1.4	4.2	32.4	32.1	29.9

**Social**	Social life	0.6	4.2	29.3	34.9	31.0
	Romantic relationships	0.8	3.6	34.1	29.3	32.1
	Smiling	0.0	6.4	23.5	31.6	38.6
	Work	0.6	4.5	47.2	26.0	21.8
	Finance	0.8	18.4	44.1	19.3	17.3

Overall, more men reported good or very good effects of oral health on different quality of life attributes compared to women, though these were not statistically significant (Table 2). However, more women (67%) reported a good/very good effect of oral health on sleep compared to men (45%). This association remained significant even after adjusting for age, ethnicity, employment and education. The adjusted odds ratio of reporting a good/very good effect by women was 2.24 (95% CI: 1.41, 3.57) compared to men.

**Table 2:** Attributes of the "effect" dimension of oral health related quality of life between sexes of study populations (n = 356).

		**Response (percent) to "effects" questions**					
		
		**Men (n = 161)**			**Women (n = 195)**		
		
**Domain**	**Attribute (effect on)**	**Very bad/Bad**	**None**	**Very good/Good**	**Very bad/Bad**	**None**	**Very good/Good**
**Physical**	Eating	8.1	18.6	73.3	11.7	18.3	70.1
	Appearance	3.7	21.1	75.2	3.6	25.4	71.1
	Speech	3.7	24.2	72.0	3.0	28.4	68.5
	General Health	3.7	24.2	72.0	4.6	20.3	75.1
	Breath	6.8	28.6	64.6	7.1	30.5	62.4
	Comfort/Relaxation	8.1	36.0	55.9	10.2	27.4	62.4

**Psychological**	Sleep	5.6	49.1	45.3	6.6	26.4	67.0^a^
	Confidence	9.3	24.8	65.8	5.1	33.5	61.4
	Worry	6.2	42.9	50.9	3.6	41.1	55.3
	Mood	6.2	46.6	47.2	7.1	42.6	50.3
	Personality	5.0	32.3	62.7	6.1	32.5	61.4

**Social**	Social life	5.0	24.8	70.2	4.6	33.0	62.4
	Romantic Relationship	5.0	35.4	59.6	4.1	33.0	64.9
	Smiling	6.2	24.8	68.9	6.6	22.3	71.1
	Work	5.6	46.0	48.4	4.6	48.2	47.2
	Finance	19.9	49.1	31.1	18.8	40.1	41.1

^a^Statistically significantly different between men and women (P < 0.05)

Table 3 shows participants' response to effect questions classified by previous visits to a dentist. A substantial proportion of those who had never visited a dentist reported good or very good effect of OHRQoL especially for the effect on eating, speech, worry, mood, and finance. This was statistically significant. The differences for effects on appearance, comfort and relaxation, work, and finance were substantial and came very close to being statistically significant (p-values ranged between 0.05–0.07). Upon multivariable adjustment for age, sex, employment, ethnicity and education, the effects on eating, relaxation, and worry remained statistically significant.

**Table 3:** Attributes of effect attributes of oral health related quality of life of participants with and without a dental visit.

		**Response (percent) to effects dimension**				
		
		**Never Visited Dentist n = 79**			**Last visit >1 year ago n = 185**	**Last visit within 1 year n = 92**
		
**Domain**	**Attribute (effect on)**	**Very bad/Bad**	**None**	**Very good/Good**	**Very good/Good**	**Very good/Good**
**Physical**	Eating	6.2	15.0	78.8	73.7	60.9 ^b^
	Appearance	3.8	15.0	81.2	71.0	69.6 ^c^
	Speech	2.5	17.5	80.0	68.3	65.2 ^b^
	General Health	3.8	20.0	76.2	75.8	67.4
	Breath	7.5	23.8	68.7	66.1	53.3
	Comfort/Relaxation	2.5	35.0	62.5	62.4	51.1 ^c^

**Psychological**	Sleep	5.0	27.5	67.5	53.2	56.5
	Confidence	10.0	15.0	75.0	55.4	69.6
	Worry	5.0	33.8	61.2	53.2	46.7 ^b^
	Mood	8.8	33.7	57.5	48.4	42.4 ^b^
	Personality	7.5	23.8	68.7	61.3	57.6

**Social**	Social life	7.5	25.0	67.5	67.2	62.0
	Romantic Relationship	7.5	33.8	58.7	64.0	58.7
	Smiling	7.5	21.2	71.2	69.4	70.7
	Work	5.0	38.8	56.2	46.2	43.5 ^c^
	Finance	0.0	53.8	46.2	34.4	32.6 ^b^

^b^Statistically significantly different compared to those reporting very good/good, (P < 0.05) but never visited a dentist; ^c^Close to being significant at the 0.05 level.

Table 4 shows the response to impact dimension of oral health on QoL among the participants in the study. Overall, a large proportion of participants reported that oral health had no impact on their daily (54%) or social activities (64%) or in talking to other people (75%). The response pattern was similar between men and women except that a substantial proportion (48%) of women reported 'little" effect on daily activities compared to 34% men.

**Table 4:** Attributes of the "impact" attributes of oral health related quality of life of study populations (n = 356).

		**Response (percent) of impact dimension**				
		
**Group**	**Domain/attributes**	**Extreme**	**Great**	**Moderate**	**Little**	**none**
**Total (n = 356)**	**Daily activities**	1.1	2.8	0.8	41.7	53.6
	**Social activities**	0.8	3.6	1.7	30.5	63.7
	**Talking to people**	0.6	2.8	0.8	20.4	75.4

**Men (n = 161)**	**Daily activities**	1.2	3.1	1.2	34.2	60.2
	**Social activities**	0.6	3.7	2.5	27.9	65.2
	**Talking to people**	0.6	3.7	0.6	21.7	73.3

**Women (n = 195)**	**Daily activities**	1.0	2.5	0.5	47.7	48.2
	**Social activities**	1.0	3.0	1.0	32.5	62.4
	**Talking to people**	0.5	2.0	1.0	19.3	77.2

The overall multivariable ANOVA model that included age, visits to a dentist, sex, education, ethnicity and employment, did not suggest statistically significant differences between groups (F = 1.22 Pr>F = 0.2788). However, a model including sex and education only suggested between group differences (F = 3.49, Pr>F = 0.0315). In this model, type-3 p-values (variables added last test) for education and sex were 0.0109 and 0.4927 respectively. Therefore we finalized our ANOVA model to one containing education only (F = 6.52, pr>F = 0.0111). The mean of effects sum score for those with secondary/tertiary education (mean = 61.8; 95% CI: 60.5, 62.9) was significantly higher than those with less than secondary/tertiary level of education (mean = 57.2; 95% CI: 57.2, 60.6).

## Discussion

To the best of our knowledge, this study is the first attempt at providing some insights into how adults in Nigeria perceive the effect of oral health on their QoL. However, this study may have limitations that may influence its interpretation and generalizability arising from the use of a convenience sample that does not represent the Nigerian adult population. An earlier version of the OHQoL-UK^© ^[13] instrument was used for this study. The effects sections of both instruments were the same, but the impact section of the current OHQoL-UK^© ^had more questions than the version used in this study. However, the main analyses in this study were made around the effects attributes of OHQoL-UK^©^.

Most of the study participants had secondary/tertiary education. This could have occurred because one of the study sites was within a university teaching hospital community. Anecdotal evidence suggests that there is difficulty in getting participants with primary education to participate in oral health questionnaire survey in these communities, especially when they perceive that they are not particularly at risk for dental disease. A study conducted in Nigeria assessing the association of socio-demographic factors and edentulism in an adult population had mostly tertiary educated participants. [34] The authors stated that one of the possible reasons for the high numbers of tertiary educated participants in their study could be because these groups of people are more informed about their oral health needs and are also more likely to seek dental treatment. [34]. In addition, tertiary-educated persons are expected to be able to afford dental service, should have better access to adequate dental care, and have better than average oral health habits. [34]

Results from this study were similar to what was reported from the study on the perception of dental esthetics between students from Nigeria and the United States of America. [25] Most participants in this study felt that their oral health had an effect, mostly a good or very good effect on their QoL, similar to earlier studies from developed countries. [34,35] Studies conducted in Nigeria have also reported that there appear to be a shortage in the number of practicing dentists, [36] and the involvement of physicians untrained in oral health care providing dental services. [28] Another study reported poor oral care and poor oral health awareness/knowledge in their study population. [29] Despite, these results study participants still rated their oral health as having an affect on their Qol.

Intriguingly, a larger proportion of participants who had never visited a dentist rated the effects of oral health on their QoL as very good/good compared to those who had visited a dentist either within the past year or earlier, or both. A possible explanation for this paradoxical observation could lie in the oral healthcare seeking behavior of Nigerian adults. If people generally visit a dentist only if there is a severe oral condition requiring immediate attention as reported in our earlier study [26] then those visiting a dentist would be an orally less healthy group compared to those who never visited a dentist. This latter group would have a better self-perceived oral health status, and consequently report higher OHRQoL attributes. This phenomenon, which we term "healthy person non-visitor effect", is perhaps similar in conceptual moorings to the well-known "healthy volunteer effect".

Observations from this study are suggestive of the "healthy person non-visitor effect" because those who have never been to a dentist consider themselves not in need of oral health care and perceive their oral health to be better and therefore do not see the need to visit a dentist. The fact that a large proportion of participants reported that their visiting a dentist depends upon a perceived need for treatment also lends credence to this idea. Another possible explanation for reporting very good/ good effect of oral health on QoL by those who have never visited a dentist may be derived from how they prioritize oral health in comparison to general health. Such self-reports could have resulted from treatment-need which is based on oral health care seeking behavior that imparts a false perception of good oral health status.

If this were to be true then a visit to a dentist would tend to lower self-rated oral health status because the person would become aware of oral problems which they were originally unaware of and could lead to fewer of such participants rating their oral health or its effects highly. In either case, this self rating of effect of oral health on QoL appears to be distal to fundamental attitudes guiding oral health seeking behavior. This study suggests a situation of low oral health awareness and a treatment need based health care seeking behavior. Okunseri *et al*. [26] reported that majority (88%) of the study participants could not afford dental treatment and 89% were not ready to spend money on it. [26] It therefore appears that the general oral health perception could be reported as "good", implying no self-perceived need for dental treatment, by default.

To support this argument, results from the study of the impact dimensions of oral health on QoL shows that more than 90% participants reported little or no impact of oral health in their daily activities, social activities or in their ability to talk to people. It has been reported that low dental care utilization was determined by age, sex and employment in the same group of participants suggesting that normative oral health care needs could be much higher than perceived needs. [26] In a study population of predominantly young persons with low oral healthcare utilization, high self perceived oral health status, poor oral health seeking behaviors observed here, the low impact attributed to oral health can be interpreted as resulting more from cultural and attitudinal factors than from an inherently healthy cohort.

Despite several limitations mentioned in this study, we have been able to describe important attributes of oral health seeking behavior and oral health quality of life factors of adults living in Benin City, Edo State, Nigeria. This study also identified some potential health care-seeking issues that might be important when considering how to promote oral health awareness and oral healthcare seeking behavior. The study could also be used by policymakers as a framework to develop appropriate oral health strategies to improve and maintain the oral healthcare of adults.

## Conclusion

This study shows that while participants reported very good/good effects of oral health on their quality of life, they also reported that oral health had little/no impact on their QoL. This, along with low oral healthcare utilization, and treatment-oriented healthcare seeking behavior could reflect cultural attributes of the population.

## Competing interests

The author(s) declare that they have no competing interests.

## Authors' contributions

CO: conceived of the study, participated in study design, carried out the study, participated in the statistical analysis, writing and in the reviewing and in responding to all reviewers' queries.

AC: set up manuscript idea, performed the statistical analysis, participated in the writing, reviewing and responding to reviewers queries.

IL: participated in the writing, critique and reviewing

CM: conceived of the study, participated in study design, writing, critique and reviewing

All authors read and approved the final manuscript.

## Pre-publication history

The pre-publication history for this paper can be accessed here:


